# Interactions of naturally occurring compounds with antimicrobials

**DOI:** 10.1016/j.jpha.2023.09.014

**Published:** 2023-09-23

**Authors:** Izabela Malczak, Anna Gajda

**Affiliations:** Department of Pharmacology and Toxicology, National Veterinary Research Institute, Partyzantów 57, 24-100, Poland

**Keywords:** Interactions, Antimicrobials, Natural compounds, Antibiotics

## Abstract

Antibiotics are among the most often used medications in human healthcare and agriculture. Overusing these substances can lead to complications such as increasing antibiotic resistance in bacteria or a toxic effect when administering large amounts. To solve these problems, new solutions in antibacterial therapy are needed. The use of natural products in medicine has been known for centuries. Some of them have antibacterial activity, hence the idea to combine their activity with commercial antibiotics to reduce the latter's use. This review presents collected information on natural compounds (terpenes, alkaloids, flavonoids, tannins, sulfoxides, and mycotoxins), of which various drug interactions have been observed. Many of the indicated compounds show synergistic or additive interactions with antibiotics, which suggests their potential for use in antibacterial therapy, reducing the toxicity of the antibiotics used and the risk of further development of bacterial resistance. Unfortunately, there are also compounds which interact antagonistically, potentially hindering the therapy of bacterial infection. Depending on its mechanism of action, each compound can behave differently in combination with different antibiotics and when acting against various bacterial strains.

## Highlights

• Naturally occurring compounds can improve the antibacterial effect of antimicrobials.
• Natural compounds can also impair the antibacterial effects of antimicrobials.
• These compounds may affect the pharmacokinetics of antimicrobials.
• These compounds may also inhibit bacterial resistance mechanisms.

## Graphical abstract

**Figure:**
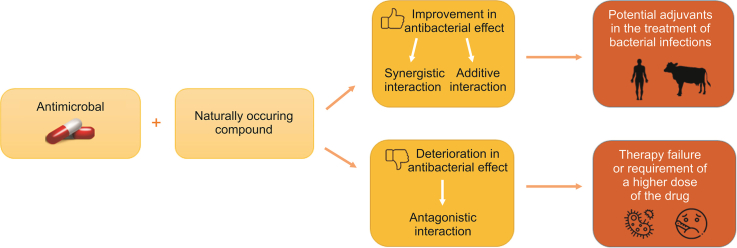

## 1 Introduction

Antibiotics are substances that, in low concentration, inhibit the growth of microorganisms or act on them bactericidal. They are produced naturally by certain bacteria and fungi, but now also synthetically. The first antibiotic, penicillin, was discovered by Alexander Fleming in 1928. Centuries before his discovery, the ancient Egyptians used mouldy bread in wounds to prevent them from getting infected [1]. Nowadays, antibiotics are also some of the most heavily used medications worldwide, and their usage has rapidly increased in past years. Between 2000 and 2018, global antibiotic consumption rates increased by 46%, even more, when only low- and middle-income countries are considered (76%) [2,3]. The application of antibiotics in agriculture is also extensive. In 2010, global antibiotic consumption was estimated at more than 63,000 tons in food animal production alone, and it is projected to rise by 67% by 2030. Two-thirds of this increase is due to the growing number of animals raised for food production [4]. Antibiotic administration is being on an immense scale, but there are many cases of their use that are not justified. The unjustified use of antibiotics is misuse, and this has negative effects. The first problem is antibiotic resistance, which many people consider as one of the greatest threats to human health [5].

Even Alexander Fleming, who, as was mentioned before, discovered penicillin, said in 1945 in a New York Times article that “… *the microbes are educated to resist penicillin and a host of penicillin-fast organisms is bred out …. In such cases, the thoughtless person playing with penicillin is morally responsible for the death of the man who finally succumbs to infection with the penicillin-resistant organism. I hope this evil can be averted*” [6]. Unfortunately, Fleming's fears have come true because of excessive use for many years, and not only do penicillin-resistant bacteria pose a real problem, but pan-resistant bacteria exist. Especially extremely dangerous are drug-resistant Gram-negative bacteria, such as carbapenemase (producing *Klebsiella pneumoniae* (*K. pneumoniae*)) or extended-spectrum β-lactamase (ESBL) (producing *Enterobacteriaceae*, *Pseudomonas aeruginosa* (*P. aeruginosa*), and *Acinetobacter baumannii* (*A. baumannii*)) [7]. People may wonder whether antibiotic-resistant bacteria are dangerous; however, the deaths of more people every year in the United States from infections with methicillin-resistant *Staphylococcus aureus* (MRSA) than from emphysema, HIV/AIDS, Parkinson's disease and homicide combined [8] ought to convince doubters. Worldwide in only 2019, bacterial antimicrobial resistance caused 1.27 million deaths and was associated with nearly 5 million [9]. Resistance also raises the cost of treating people suffering from infections caused by these bacteria. In Europe alone, it is estimated that antimicrobial resistance requires treatment expenditure of more than 9 billion euros annually. In the United States, according to the Centers for Disease Control and Prevention, it adds around 20 billion dollars to healthcare costs and is an even larger financial burden if the cost to society of lost productivity is considered [10]. The misuse of antibiotics on farms also greatly impacts the development of bacterial resistance. Depending on the pharmacokinetics of antibiotics used in animals, they can pass with urine or feces into soil or water, disturbing the microbiome of that environmental compartment. In addition, humans can also become infected with resistant bacteria from such animals, mainly through direct contact or through the food chain [11].

The second issue is the side effects. Of course, antibiotics used in human and veterinary medicine are safe, especially if properly administered, but the increasing doses needed to achieve therapeutic success can have serious and health-threatening consequences. One of the undesirable effects is the development of an allergic reaction to antibiotics, which can be life-threatening in the case of a very strong anaphylactic shock reaction [12].

Another challenge may be their toxic effects, such as hepatotoxicity or nephrotoxicity, which may be dangerous to human or animal health over long-term antibiotic use [13,14].

The occurrence of antibiotic residues in the food chain is also disturbing. Improper use of antimicrobial therapy or not taking standard precautions during the withdrawal period can lead to accumulating such residues [15]. These pose a real threat to the health of the consumer. The residues can transfer antibiotic-resistant bacteria and exert immunopathological or harmful effects on various organs in the human body [16].

A final issue, which may not be as important as others but still worth considering, is that over the last decade, the number of new antibiotics developed has decreased significantly [17]. Consequently, there are insufficient alternative new treatments, which is worrying given the emergence of increasingly resistant bacteria.

To resolve these issues, new solutions must be found to complement or replace traditional infection treatment regimens. Compounds of plant origin from selected groups have proven in many studies that their antibacterial activity can be helpful [[18], [19], [20], [21]]. However, better effectiveness of plant-origin drugs might be attained by their administration in combination with drugs that are already used, which would allow the dose of the antibiotic to be reduced. This review aims to present compounds from terpenes, alkaloids, flavonoids, tannins, sulfoxides, and mycotoxins that could potentially be used as adjuvants in treating bacterial infections, and additionally aims to identify interactions between them and antibiotics. The interactions of antibiotics with other compounds and the study of these interactions have history almost as rich as antibiotics themselves [22]. It has also been known for a long time that the substances we consume, which are often part of our food, can also affect the effectiveness of the medicinal preparations we take. The collected work presents a cross-section of knowledge from approximately the last 20 years on potential interactions between antimicrobials and compounds found in nature [23].

## 2 Antibacterial effect of plant-derived compounds

Plants can produce aromatic substances, of which at least 12,000 of the 120,000 predicted to exist had already been isolated at the end of the 20th century [24]. These compounds usually play a defensive role in plants against other various organisms. Some cause a characteristic odour in plants; some are responsible for their colour, and others for their taste.

Plants have been used in medicine for a very long time, and in some countries, they are still an indispensable element of health care. In African countries, a good example is the chewing stick used in oral hygiene. The chewing stick can be made from a variety of plants, but the ones that have been tested contain antibacterial compounds. In India, the healing craft known as Ayurveda is practised using plant extracts individually or in mixtures. Also, in many studies, researchers have proved that compounds of plant origin, mainly their secondary metabolites, have antibacterial activity. These compounds belong to chemical groups such as tannins, alkaloids, terpenes and flavonoids [25].

Compounds of plant origin may exhibit antibacterial activity using various mechanisms, such as disruption of membrane function and structure, interruption of DNA/RNA synthesis and function, and interference with metabolism or the interaction of normal cell communication [26]. Another important mechanism in the context of antibacterial therapy is the ability of plant secondary metabolites to interfere with the production of toxins by bacteria. Essential oils of clove, thyme, cinnamon, and isolated eugenol reduced the production of listeriolysin O toxin by *Listeria monocytogenes* (*L. monocytogenes*) bacteria [27]. On the other hand, carvacrol inhibited the production of toxins by *Bacillus cereus* and *Clostridium difficile* [28,29]. An important ability of plant-origin compounds is to counter the defence mechanisms of bacteria against antibiotics. One of those mechanisms is the production of various efflux pumps by bacteria, which release substances harmful to other bacteria outside the bacterial cell. The occurrence of this phenomenon leads to multi-drug resistance. Therefore, inhibitors of these efflux pumps are sought, which would make it possible to reverse bacterial resistance to some antibiotics. Studies have already registered discoveries of compounds capable of inhibiting the bacterial efflux pump among plant metabolites. Such compounds include berberine, gallic acid, or capsaicin, which have an inhibitory effect on the NorA efflux pump of *Staphylococcus aureus* (*S. aureus*). Unfortunately, despite finding drug candidates, this concept is still not used in therapy [30].

## 3 Characteristics of interactions between compounds

Articles focusing on interactions between two or more compounds make up a huge part of the research in the biomedical science field. For example, the phrases “drug interactions” and “drug combinations” had more than 5 million results in a search through the literature. However, it is not easy to give one exact definition of an interaction or one method to determine the occurrence of one [31]. In a Review article by Greco et al. [32], 13 different methods are described for determining synergy alone.

Nevertheless, it is accepted that interactions can be classified as synergistic, additive, or antagonistic. In general, when thinking about synergism, what is meant is an interaction between two or more compounds in which the total effect of these compounds exceeds the sum of their individual effects. An additive effect is the combined effect of two or more chemicals equal to the sum of the impact of each agent given alone; this type of interaction is usually exploited to reduce a specific adverse effect of a drug. Antagonistic interaction means that the aggregate impact is less than the original effect of the tested compound administered separately, which may result in treatment failure in a case of a drug interaction.

Different experimental approaches can be seen in other studies, making comparing the results between experiments impossible. The most commonly used include the diffusion assay, the checkerboard method, the isobole method, and the time-kill assay [33]. A term often used in antimicrobial interaction studies is the fractional inhibitory concentration (FIC) index (FICI), the sum of the FICs of each compound tested when combined. This concentration is calculated by dividing each drug's (compound) minimum inhibitory concentration (MIC) when combined with its MIC when used alone. A FICI quantifies drug/compound interactions and uses the value to categorize the interactions between substances. The FICI values used to interpret the results are ≤0.50 defining synergy, >0.50 to ≤1.00 representing an additive effect, >1.00 to ≤2.00 signifying no net effect, and >2.00 manifesting antagonism [34].

The methods described above are used to study the interaction of compounds with antimicrobials in vitro and are based on comparisons of the MICs of the combinations used; however, the effectiveness of therapy is not only affected by the sensitivity of the bacteria to a given preparation but also by that preparation's bioavailability. Therefore, some compounds can also interact with antimicrobials by changing their pharmacokinetic properties and not only by increasing the sensitivity of bacteria.

## 4 Compounds with proven interactions

### 4.1 Terpenes

Terpenes are a class of natural products usually obtained from plants. Their broad diversity in chemical structures casts their various roles in the interaction between organisms. For example, plants can use terpenes to attract pollinating insects or, conversely, to repel potential predators by their odour. Terpenes can also act as signal transducers and phytohormones in plants. Many insects metabolize the terpenes they obtain from ingested plant material to produce hormones and pheromones [35]. They are formed in either the mevalonic acid or methylerythritol phosphate pathways, as these two pathways supply isopentenyl diphosphate and dimethylallyl diphosphate, which are the universal building blocks of terpenes [36]. It has been observed that terpenes and their derivatives have many useful properties, for example, some of which are anti-inflammatory [37,38], antioxidant [39,40], antifungal [41], or antibacterial [[42], [43], [44]]. The studies also suggest that some of the compounds may also be effective in increasing the activity of antibiotics in treating bacterial infections. The tested combinations of terpenes and antimicrobials are summarized and presented in Table 1 [[45], [46], [47], [48], [49], [50], [51], [52], [53], [54], [55]].

**Table 1:** Combinations of terpenes with antimicrobial agents.

Combinations of terpenes with antimicrobial agents	Compounds naturally occurring in plants	Antimicrobial	Bacteria species	Refs.
Combinations of terpenes with antimicrobial agents resulting in minimum inhibitory concentration (MIC) reduction	Thymol	Gentamicin	*Klebsiella pneumoniae*	[45]
			*Pseudomonas aeruginosa*	[45]
			*Staphylococcus aureus*	[45]
		Neomycin	*Staphylococcus aureus*	[45]
		Penicillin G	*Streptococcus mutans*	[45]
		Ceftriaxone	*Enterococcus faecalis*	[45]
		Vancomycin	*Escherichia coli*	[46]
		Azithromycin	*Pythium insidiosum*	[47]
		Clarithromycin	*Pythium insidiosum*	[47]
		Minocycline	*Pythium insidiosum*	[47]
		Tigecycline	*Pythium insidiosum*	[47]
		Tetracycline	*Staphylococcus aureus*	[48]
		Tilmicosin	*Pasteurella multocida*	[49]
			*Mannheimia haemolytica*	[49]
		Doxycycline	*Pasteurella multocida*	[49]
			*Mannheimia haemolytica*	[49]
	Carvacrol	Azithromycin	*Pythium insidiosum*	[47]
		Clarithromycin	*Pythium insidiosum*	[47]
		Minocycline	*Pythium insidiosum*	[47]
		Tigecycline	*Pythium insidiosum*	[47]
		Tetracycline	*Staphylococcus aureus*	[48]
		Bacitracin	*Listeria monocytogenes*	[50]
			*Listeria innocua*	[50]
		Erythromycin	*Listeria monocytogenes*	[50]
			*Listeria innocua*	[50]
			Group A *Streptococcus* spp.	[51]
		Colistin	*Listeria monocytogenes*	[50]
			*Listeria innocua*	[50]
		Tilmicosin	*Pasteurella multocida*	[49]
			*Mannheimia haemolytica*	[49]
		Doxycycline	*Pasteurella multocida*	[49]
			*Mannheimia haemolytica*	[49]
	Citral	Bacitracin	*Listeria monocytogenes*	[50]
			*Listeria innocua*	[50]
		Erythromycin	*Listeria monocytogenes*	[50]
			*Listeria innocua*	[50]
		Colistin	*Listeria monocytogenes*	[50]
			*Listeria innocua*	[50]
	Amyrin	Vancomycin	*Staphylococcus aureus*	[52]
	Betulinic acid	Vancomycin	*Staphylococcus aureus*	[52]
	Betulinaldehyde	Vancomycin	*Staphylococcus aureus*	[52]
	Amyrin	Methicillin	*Staphylococcus aureus*	[52]
	Betulinic acid	Methicillin	*Staphylococcus aureus*	[52]
	Betulinaldehyde	Methicillin	*Staphylococcus aureus*	[52]
	12(*S*),16ξ-dihydroxycleroda-3,13-dien-15,16-olide	Meropenem	*Staphylococcus aureus*	[53]
		Oxacillin	*Staphylococcus aureus*	[53]
	16α-hydroxycleroda-3,13(14)-*Z*-dien-15,16-olide	Norfloxacin	*Staphylococcus aureus*	[54]
		Ciprofloxacin	*Staphylococcus aureus*	[54]
		Ofloxacin	*Staphylococcus aureus*	[54]
Combinations of terpenes with antimicrobial agents resulting in MIC increase	Citral	Ciprofloxacin	*Pseudomonas aeruginosa*	[55]
		Chloramphenicol	*Pseudomonas aeruginosa*	[55]
		Tobramycin	*Pseudomonas aeruginosa*	[55]
		Gentamicin	*Pseudomonas aeruginosa*	[55]
		Colistin	*Pseudomonas aeruginosa*	[55]

#### 4.1.1 Thymol

Thymol is a monoterpenoid phenol found naturally in oil extracted from *Thymus vulgaris* L., commonly known as thyme, and other plants such as *Ocimum gratissimum* L., *Carum copticum* L., *Oliveria decumbens* Vent, different species of the genera *Origanum* L., *Satureja* L., and many others [56].

Thymol combined with aminoglycosides reduced the MIC of the antibiotic considerably, thereby reducing the necessary dose needed for therapeutic success. Thymol in subinhibitory concentrations (MIC/8) showed an improvement in antibiotic activity against *K. pneumoniae* and *P. aeruginosa* infections combined with gentamicin, reducing MICs 32 and 4 times, respectively. This compound also improved performance with gentamicin and neomycin when used against *S. aureus*, resulting in a 16-fold and 32-fold decrease in MIC, respectively. As for β-lactams, thymol significantly reduced the penicillin G MIC from 128 to 2 μg/mL when this combination was used against *Streptococcus mutans*, and lowered the ceftriaxone MIC for *Enterococcus faecalis* from 64 to 4 μg/mL [45]. This monoterpenoid (MIC/4), in combination with ethylenediaminetetraacetic acid (EDTA) (MIC/4) and vancomycin, showed high activity against Gram-negative *Escherichia coli* (*E. coli*), increasing the sensitivity of this bacteria to the antibiotic 16-fold [46]. It is a particularly useful interaction considering that Gram-negative bacteria are not susceptible to the action of vancomycin because of their outer bacterial membrane, which prevents the penetration of antibiotics into the cell [57]. In in vitro studies using *Pythium insidiosum* as the infectious agent, both thymol (1.25–80 μg/mL) and carvacrol (1.25–40 μg/mL) were synergistic in combinations with azithromycin, clarithromycin, minocycline, and tigecycline in checkerboard test. The combinations of these terpenes with azithromycin, clarithromycin, and minocycline have proven particularly effective since they demonstrated synergies from 60% up to 92% (for the clarithromycin and thymol interaction) based on the mean FICI [47].

#### 4.1.2 Carvacrol

Carvacrol (2-methyl-5-(1-methylethyl)-phenol) is a monoterpenic phenol, isomeric with thymol, and found in many aromatic plants including *Origanum dictamnus*, *Origanum majorana,* and *Origanum vulgare, Satureja hortensis* and *Satureja montana*, *Thymbra capitata*, and *Thymus serpyllum, Thymus vulgaris,* and *Thymus zygis* [58].

Interaction with antibiotics was shown in research containing essential oil from *Origanum vulgare* and its main compounds, thymol, and carvacrol, where it was proved that the compounds enhanced the inhibitory effects of tetracycline against *S. aureus*. Both terpenes reduced, in subinhibitory concentrations (MIC/4), the MIC of tetracycline from 64 to 32 μg/mL. The mechanism responsible for this decrease in MICs is probably inhibiting efflux pumps in bacterial membranes [48]. Another mechanism responsible for the synergistic action of thymol and carvacrol with the antibiotics mentioned above may be their ability to increase the cellular permeability of bacteria due to the lipophilic effect of these terpenes on the cell membrane [59,60]. Carvacrol added to erythromycin synergistically impacts 21 out of 32 strains of group A streptococci resistant to this antibiotic, while no interactions were observed for the remaining 11 strains [51]. An antibiotic in the tetracycline family, doxycycline, has also been investigated in its interaction with carvacrol for potential therapeutic efficacy against bovine respiratory disease. Although the aetiology of the disease is not only bacterial infection, *Pasteurella multocida* and *Mannheimia haemolytica* bacteria are important factors in its development. The primary treatments for this condition are doxycycline and tilmicosin. Combining carvacrol with doxycycline showed a synergistic effect in treating bovine respiratory disease caused by *Pasteurella* *multocida* and *Mannheimia* *haemolytica* [49]. The other primary antibiotic for therapy of this disease, the macrolide tilmicosin, rarely encounters bacterial resistance, but research found extra pathways leading to macrolide resistance by these bacteria [61]. When tested with *Pasteurella* *multocida* and *Mannheimia* *haemolytica*, tilmicosin gave an additive effect in combination with carvacrol and thymol, as did thymol in combination with doxycycline [49].

#### 4.1.3 Citral

Citral is an acyclic monoterpene aldehyde, a mixture of two geometric isomers, geranial and neral. It can be found in *Pelargonium crispum*, *Cymbopogon citratus,* and *Citrus limon* [62].

In a study investigating interactions in combatting infection by two species of Gram-negative *Listeria* bacteria, citral (0.150 and 0.250 μg/mL) was also used besides carvacrol (0.100 and 0.175 μg/mL). Of the antibiotics administered in listeriosis treatment, i.e., erythromycin, bacitracin, and colistin, only erythromycin is active against both *L. monocytogenes* and *Listeria innocua*. However, using terpenes in the medium made it possible to lower the MIC of all antibiotics for both bacterial species. For example, carvacrol reversed the bacterial resistance to bacitracin. The best results were obtained with a mixture of carvacrol (0.100 μg/mL) and citral (0.150 and 0.250 μg/mL) in a medium that reversed bacterial resistance to bacitracin and colistin [50].

On the other hand, research has provided evidence for the potential risk of using citral with some antibiotics. Exposure of *P. aeruginosa* to citral (2 or 4 mg/mL) caused an increase in the MICs of ciprofloxacin, chloramphenicol, tobramycin, gentamicin, and colistin, through mechanisms such as overexpression of resistance-nodulation-division efflux operons, changes in zeta potential or formation of inactive citral-based adducts over time [55].

#### 4.1.4 Pentacyclic triterpenoids

Pentacyclic triterpenoids are secondary plant metabolites in fruit peel, leaves, and stem bark. They are biologically active phytochemicals with anti-inflammatory, hepatoprotective, antihypertensive, antiulcerogenic, and antitumorigenic activities [63,64].

Research showed synergistic antimicrobial activity between pentacyclic triterpenoids and antibiotics against methicillin-sensitive *S*. *aureus* (MSSA) and MRSA strains. The terpenes tested were α-amyrin, betulinic acid, and betulinaldehyde in concentration from 0.25 to 64 μg/mL, and the tested antibiotics were vancomycin and methicillin. Betulinaldehyde only had a synergistic effect against a reference strain of MSSA when in combination with methicillin, and betulinaldehyde enhanced the antibacterial activity of methicillin for both MRSA and MSSA strains and was used with vancomycin. The MIC of the antibiotic was reduced when acting against both strains. Better results were obtained with α-amyrin and betulinic acid because they showed synergistic effects, with a FICI of ≤0.5 in combination with both antibiotics tested in this research. The strongest synergic interaction occurred when betulinic acid was combined with methicillin, which resulted in a 64-fold MIC reduction. Betulinic acid also interacted synergistically with vancomycin, reducing the MIC eightfold [52].

#### 4.1.5 Clerodane diterpenes

The clerodane diterpenes are bicyclic diterpenes and a large group of secondary metabolites found in several hundred plants [65]. One isolated from *Callicarpa americana*, 12(*S*),16ξ-dihydroxycleroda-3,13-dien-15,16-olide, potently synergized with oxacillin and meropenem against MRSA, making it useful in the treatment of β-lactam-resistant infections [53]. Another study showed that clerodane diterpene 16α-hydroxycleroda-3,13(14)-*Z*-dien-15,16-olide (100–0.78 μg/mL) from the leaves of *Polyalthia longifolia* also had a synergistic effect when combined with the fluoroquinolones norfloxacin, ciprofloxacin, and ofloxacin [54].

### 4.2 Alkaloids

Alkaloids are basic compounds naturally occurring in plants and contain one or more nitrogen atoms (usually in a heterocyclic ring). They usually have pharmacological effects when administered to humans or animals [66], and several authors recognized that one of these effects might be antimicrobial [[67], [68], [69], [70]]. This led researchers to the idea that they might also interact helpfully with drugs currently used to treat bacterial infections. The combinations of alkaloids and antimicrobials tested and showing promise are summarized in Table 2 [[71], [72], [73], [74], [75], [76], [77], [78], [79], [80], [81], [82], [83], [84], [85], [86], [87]].

**Table 2:** Combinations of alkaloids with antimicrobial agents.

Combinations of alkaloids with antimicrobial agents	Compounds naturally occurring in plants	Antimicrobial	Bacteria species	Refs.
Combinations of alkaloids with antimicrobial agents resulting in MIC reduction	Capsaicin	Ciprofloxacin	*Staphylococcus aureus*	[71]
	Piperine	Rifampicin	*Staphylococcus aureus*	[72]
			*Pseudomonas aeruginosa*	[72]
		Tetracycline	*Staphylococcus aureus*	[72]
		Streptomycin	*Klebsiella pneumoniae*	[73]
			*Salmonella enterica*	[74]
		Kanamycin	*Klebsiella pneumoniae*	[73]
			*Salmonella enterica*	[74]
		Amikacin	*Klebsiella pneumoniae*	[73]
			*Salmonella enterica*	[74]
	Berberine	Tigecycline	*Acinetobacter baumannii*	[75]
		Sulbactam	*Acinetobacter baumannii*	[75]
		Meropenem	*Acinetobacter baumannii*	[75]
		Ciprofloxacin	*Acinetobacter baumannii*	[75]
			*Klebsiella pneumoniae*	[76]
		Linezolid	*Staphylococcus* spp.	[77]
			oral *Streptococcus* strains	[78]
		Cefoxitin	*Staphylococcus* spp.	[77]
		Erythromycin	*Staphylococcus* spp.	[77]
			oral *Streptococcus* strains	[78]
		Penicillin G	oral *Streptococcus* strains	[78]
		Clindamycin	oral *Streptococcus* strains	[78]
	Tetrandrine	Cefazolin	*Staphylococcus aureus*	[79]
	Demethyltetrandrine	Cefazolin	*Staphylococcus aureus*	[79]
	Sanguinarine	Ampicillin	*Staphylococcus aureus*	[80]
		Oxacillin	*Staphylococcus aureus*	[80]
		Norfloxacin	*Staphylococcus aureus*	[80]
		Ciprofloxacin	*Staphylococcus aureus*	[80]
		Vancomycin	*Staphylococcus aureus*	[80]
		Streptomycin	*Enterococcus* spp.	[81]
			*Staphylococcus aureus*	[81]
			*Staphylococcus epidermidis*	[81]
			*Streptococcus pyogenes*	[81]
			*Streptococcus agalactiae*	[81]
			*Bacillus subtilis*	[81]
			*Acinetobacter baumannii*	[81]
			*Pseudomonas aeruginosa*	[81]
			*Klebsiella pneumoniae*	[81]
			*Escherichia coli*	[81]
	Lysergol	Nalidixic acid	*Escherichia coli*	[82]
	Squalamine	Chloramphenicol	*Escherichia coli*	[83]
			*Klebsiella pneumoniae*	[83]
			*Pseudomonas aeruginosa*	[83]
		Cefepime	*Escherichia coli*	[83]
			*Klebsiella pneumoniae*	[83]
			*Pseudomonas aeruginosa*	[83]
		Tetracycline	*Escherichia coli*	[83]
			*Klebsiella pneumoniae*	[83]
			*Pseudomonas aeruginosa*	[83]
		Ciprofloxacin	*Escherichia coli*	[83]
			*Klebsiella pneumoniae*	[83]
			*Pseudomonas aeruginosa*	[83]
	Tomatidine	Gentamicin	*Staphylococcus aureus*	[83]
		Tobramycin	*Staphylococcus aureus*	[84]
	Caffeine	Gentamicin	*Staphylococcus aureus*	[85]
		Azithromycin	*Klebsiella pneumoniae*	[85]
		Novobiocin	*Klebsiella pneumoniae*	[85]
		Amoxicillin	*Staphylococcus aureus*	[86]
		Cefepime	*Pseudomonas aeruginosa*	[85]
			*Acinetobacter baumannii*	[85]
		Kanamycin	*Proteus vulgaris*	[87]
			*Escherichia coli*	[87]
			*Bacillus cereus*	[87]
			*Shigella sonnei*	[87]
			*Plesiomonas shigellosis*	[87]
			*Klebsiella pneumoniae*	[87]
			*Enterococcus faecalis*	[87]
		Tetracycline	*Pseudomonas aeruginosa*	[87]
Combinations of alkaloids with antimicrobial agents resulting in minimum inhibitory concentration (MIC) increase	Caffeine	Novobiocin	*Staphylococcus aureus*	[85]
		Cefepime	*Staphylococcus aureus*	[85]
			*Klebsiella pneumoniae*	[85]
		Gentamicin	*Acinetobacter baumannii*	[85]
		Benzylpenicillin	*Staphylococcus aureus*	[86]
		Chloramphenicol	*Pseudomonas aeruginosa*	[87]
			*Proteus vulgaris*	[87]
			*Escherichia coli*	[87]
			*Bacillus cereus*	[87]
			*Shigella sonnei*	[87]
			*Plesiomonas shigellosis*	[87]
			*Klebsiella pneumoniae*	[87]
			*Enterobacter cloacae*	[87]
		Kanamycin	*Pseudomonas aeruginosa*	[87]
		Nalidixic acid	*Pseudomonas aeruginosa*	[87]
			*Proteus vulgaris*	[87]
			*Escherichia coli*	[87]
			*Bacillus cereus*	[87]
			*Shigella sonnei*	[87]
			*Plesiomonas shigellosis*	[87]
			*Enterobacter cloacae*	[87]
			*Enterococcus faecalis*	[87]
		Erythromycin	*Pseudomonas aeruginosa*	[87]
			*Proteus vulgaris*	[87]
			*Bacillus cereus*	[87]
			*Shigella sonnei*	[87]
			*Plesiomonas shigellosis*	[87]
			*Enterobacter cloacae*	[87]
			*Enterococcus faecalis*	[87]
		Tetracycline	*Proteus vulgaris*	[87]
			*Bacillus cereus*	[87]
			*Shigella sonnei*	[87]
			*Plesiomonas shigellosis*	[87]
			*Klebsiella pneumoniae*	[87]
			*Enterococcus faecalis*	[87]
		Metronidazole	*Pseudomonas aeruginosa*	[87]
			*Proteus vulgaris*	[87]
			*Shigella sonnei*	[87]
			*Plesiomonas shigellosis*	[87]
			*Enterobacter cloacae*	[87]
			*Klebsiella pneumoniae*	[87]
			*Escherichia coli*	[87]

#### 4.2.1 Capsaicin

Capsaicin is an alkaloid found in fruits from plants belonging to the genus *Capsicum* in amounts of up to 1.5%, the content being affected by environmental conditions and the age of the fruit. Capsaicin appears to be a suitable adjuvant candidate for antibiotic treatment, because of its antimicrobial effects [[88], [89], [90]] and its capability of increasing drug absorption, which was indicated in studies [91,92]. Capsaicin in concertation 25 mg/mL significantly reduced the MIC of ciprofloxacin for *S. aureus* and extended the post-antibiotic effects at MIC, probably by inhibiting the NorA bacterial efflux pump [71]. Another study showed that adding capsaicin (in concentrations 0.01%, 0.1%, 0.5%, and 1%) to a ciprofloxacin preparation increases this antibiotic's oral absorption, but not in a linearly progressive manner since the highest concentration of capsaicin (1%) tested had a lower effect than smaller concentrations. This phenomenon has been caused probably because higher concentrations of capsaicin may induce in certain conditions and concentrations, apoptosis of epithelial cells in the gastrointestinal tract health [93,94]. The authors did not investigate the mechanism of this phenomenon in detail, but their theory is that capsaicin increases bioavailability by increased blood perfusion at the absorption site (intestinal pilli) due to the well-known irritant effects of capsaicin. Also, another possibility is that capsaicin promotes gastric emptying and this may pass ciprofloxacin faster to the duodenum where a more favorable pH and absorption surface could facilitate drug absorption [93]. The addition of 20 μg/kg body weight of capsaicin to the preparation of enrofloxacin achieved higher maximum concentrations of the antibiotic in chicken serum after oral administration [95]. This has also been explored in studies by Komori et al. [96], where the effect of capsaicin on intestinal cefazolin absorption in rats was investigated. The cefazolin absorption in the rat jejunum was significantly increased in the presence of 10 and 400 μM capsaicin. Based on the collected information, the author speculates that the reason for the increased absorption of cefazolin may be the increased concentration of intracellular Ca^2^^+^ caused by capsaicin, which expands the tight junction of the epithelium in the intestine, and thus increases the intestinal pericellular permeability of less absorbable compounds. However, the same research group conducted other studies in which capsaicin decreased the absorption of the drug instead of increasing it. In this study, cephalexin absorption clearance was significantly decreased in the presence of 400 μM. Still, it was not altered in the presence of 10 μM, which indicates that the intestinal absorption of cefazolin via passive diffusion seems more sensitive to capsaicin than that via transporters, as in the case of cephalexin. Authors speculate that capsaicin is the reason, which stimulates the afferent neurons by associating with transient receptor potential cation channel subfamily V member 1 (TRPV1) and the neurons release hormones affecting the gastrointestinal tract such as vasoactive intestinal polypeptide (VIP). VIP modulates the Na/H exchanger on the epithelial cells and the proton gradient across the cellular membrane is altered. This affects the H^+^/peptide co-transporting activity of H^+^/peptide co-transporter 1 (PEPT1), which mediate cephalexin absorption [97].

#### 4.2.2 Piperine

Piperine is another alkaloid that appears to be useful in antimicrobial therapy. Piperine is an alkaloid found in the black pepper (*Piper nigrum*), long pepper (*Piper longum*), and the fruit of other species belonging to the family *Piperaceae*. Piperine is responsible for black pepper's distinct biting taste, but it also has many other properties, such as reduction of insulin resistance, anti-inflammatory effects, and improvement of hepatic steatosis [98].

Many existing studies in the literature have examined piperine's ability to enhance the bioavailability of antibiotics. A study by Janakiraman and Manavalan [99] concluded that co-administration of piperine (20 mg/kg body weight) with norfloxacin and ampicillin improved their oral bioavailability. Piperine may cause alterations in the permeability of gastrointestinal epithelial cells as well as suppression of enzymes involved in the conversion of ampicillin to penicilloic acid. Other research showed that oral co-administration of piperine (10 and 20 mg/kg body weight) enhanced the bioavailability of amoxicillin and cefotaxime significantly. Piperine may alter numerous physiological processes, such as changes in the gastrointestinal environment and/or the process of transport or absorption, according to the authors' analysis of several pharmacokinetic characteristics. However, because piperine has been found to inhibit microsomal enzymes and enzyme systems, they cannot rule out a direct inhibitory impact of piperine on the microsomal metabolizing enzymes involved in the metabolism of amoxicillin or cefotaxime [100]. Piperine and piperlongumine, an alkaloid derived from *Piper longum*, are capable of improving the effectiveness of rifampicin and tetracycline against *S. aureus* and the effectiveness of rifampicin against *P. aeruginosa*. A study by Mgbeahuruike et al. [72] of piperine, piperlongumine, rifampicin, tetracycline, and itraconazole interactions showed a synergistic interaction of rifampicin and piperine (5 mg/mL) but also showed that the type of obtained effect, i.e., whether the interaction was synergistic, additive, or antagonistic, was greatly dependent on the ratio of the alkaloid to the antibiotic. A study of biofilm-associated infections, alongside thymol and piperine both in concentrations between 16 and 1024 μg/mL, showed synergistic antibiofilm effects when combined with streptomycin, kanamycin, and amikacin against *K. pneumoniae* and *Salmonella enterica* [73,74].

#### 4.2.3 Berberine

Berberine is the isoquinoline-type alkaloid isolated from many plants, including members of the genus *Berberis*. In different parts of the plant *Berberis microphylla*, the alkaloids berberine, allocryptopine, calafatine, jatrorrhizine, palmatine, protopine, reticuline, thalifendine, and tetrahydroberberine can be found. A mixture of these alkaloids seemed to have promising antibacterial activity against Gram-positive bacteria and showed synergistic effects with ampicillin and cephalothin against certain bacterial strains [101]. Many studies emphasize the positive effect of berberine alone on antibacterial therapy. When this compound was injected in concentration 20 mg/kg body weight and was used against multi-drug-resistant *A*. *baumannii*, it showed weak antimicrobial activity with a MIC > 256 mg/L. Still, when it was combined with antibiotics, it dramatically increased the susceptibility of the strains to the point where it reversed their resistance to antibiotics, e.g., tigecycline, sulbactam, meropenem, and ciprofloxacin. Berberine hydrochloride was concluded to be a pump competitor because it boosted the expression of the pump gene *adeB* and had a higher affinity for AdeB efflux pump than antibiotics. That reduced extrusion of antibiotics by the AdeABC pump, which is responsible for resistance to some antibiotics [75]. The combination of ciprofloxacin and berberine (1024 μg/mL) exhibited antimicrobial effects against multi-drug-resistant *K. pneumoniae*, where a synergistic interaction was observed against 18.18% of isolates, and the concentration of ciprofloxacin could be decreased by 75% in combination with berberine. Although the mechanism of this interaction has not yet been thoroughly investigated, it has been proven that the transcription levels of the efflux pumps acrA, acrB, tolC and acrR were upregulated in strains treated with berberine and the effect was even stronger in strain treated with combination of berberine and ciprofloxacin. This may be one of the potential explanations for synergism, because in *K. pneumoniae*, acrAB-tolC dominates efflux-mediated resistance to fluoroquinolones [76]. After adding berberine to Mueller-Hinton agar medium, an expansion of the zone of inhibition of growth of coagulase-negative *Staphylococcus* strains was observed in the presence of one of ten tested antibiotics compared to the zone size in the medium without berberine. In the presence of berberine at a concentration corresponding to 1/4MIC, significantly better inhibition was observed in the growth of 7 out of 14 strains. The most susceptible strains towards berberine were *Staphylococcus* *haemolyticus* ATCC29970, *Staphylococcus* *epidermidis* ATCC 12228, *Staphylococcus* *capitis subsp. capitis* ATCC35661, *Staphylococcus* *galinarium* ATCC700401, *Staphylococcus* *hominis subsp. hominis* ATCC27844, *Staphylococcus* *intermedius* ATCC29663, and also *Staphylococcus* lugdunensis ATCC49576. In other strains, the growth inhibition was observed only to a minor extent and no antagonistic interaction was noticed. The most remarkable synergistic effect was observed in the combinations of berberine and linezolid, cefoxitin, and erythromycin applied against oral bacteria [77]. Oral *Streptococcus* strains are troublesome for immunocompromised people at high risk of opportunistic infection or tooth decay caused by streptococcal species. Berberine chloride inhibited the growth of oral pathogens, especially at concentrations above 125 mg/mL. The investigated combinations of this compound and the antibiotics caused a reduction in the viability of bacteria that was very similar to the viability reduction when active agents were used alone at higher concentrations. The combinations of berberine chloride (in concentration of MIC/4) and penicillin, clindamycin, erythromycin, and linezolid yielded the best inhibitory results on the growth zone. The smallest and non-significant differences in the growth inhibition zone sizes were found in trimethoprim and sulfamethoxazole, tetracycline, and oxacillin combinations. Only the interaction of berberine chloride and ciprofloxacin was slightly antagonistic when assayed for inhibition of *Streptococcus* *mutans* and *Streptococcus* *oralis*. It is believed that the synergistic and enhanced activity of berberine chloride with common antimicrobial agents can be attributed to suppressing or inhibiting the efflux pump of bacterial multi-drug resistance (MDR), which is responsible for removing antibacterial agents from the cell. Berberine can build up in bacteria and may prevent the MDR pump from clearing some antibiotics. Berberine may also act synergistically with antibiotics through the mechanisms of bactericidal action, which include destruction of the structure of the bacterial cell, reduction of DNA replication and/or RNA transcription, and disturbance of the stability of biofilm by inducing protein binding [78].

#### 4.2.4 Caffeine

Caffeine is a natural methylxanthine derivative that acts as an antagonist of adenosine A1, A2A, and A2B receptors and its best-known source is the coffee bean, the seed of the *Coffea* plant [102].

In disc diffusion method caffeine showed a marked enhancement of antistaphylococcal activity for five antibiotics: novobiocin, cefepime, gentamycin, azithromycin, and ticarcillin. Antibiotics with promising results were further investigated using microbroth dilution assay and checkerboard methodology. Caffeine at concentration between 250 μg/mL and 2 mg/mL was able to show a synergistic effect with gentamicin when used against *S. aureus* MRSA strains, for azithromycin and novobiocin against *K. pneumoniae*, and with cefepime against *P*. *aeruginosa* and *A*. *baumannii*. Surprisingly, in this method, novobiocin showed an antagonistic effect in combination with *S. aureus* strains and also against MRSA strains, the combination with cefepime showed antagonism. Moreover, antagonism was observed with the combination of cefepime against *K. pneumoniae* and with gentamicin in *A. baumannii*. The author, however, did not investigate what could be the potential mechanism of these interactions [85].

In another study where overlay inoculum susceptibility disc method was used to test interaction of penicillin antibiotics against *S. aureus*. Caffeine at 5 and 10 mg/mL decreased the MIC of amoxicillin by 22 and 25 times respectively and gave synergistic effect. However, it also showed an antagonistic effect because in combination with benzylpenicillin, the MIC of antibiotic increased by 59 and 40 times at caffeine concentrations of 5 and 10 mg/mL respectively. The precise mechanism of this interaction observed between caffeine and amoxicillin is unknown. However, it is possible that the inhibition of bacterial cell wall by penicillins, which results in lyses of the cells, might facilitate the influx of caffeine into the bacterial cells. Such higher concentration in the cells will enhance the damage on DNA caused by caffeine. The synergistic effect may also be as a result of the inhibition of *Staphylococcus penicillinase* enzyme, which will potentiate the activity of the penicillinase sensitive antibiotics. However, the mechanism through which benzylpenicillin and caffeine form an antagonistic interaction is also not clear [86].

Caffeine has also shown negative effects on the effectiveness of antibiotics such as chloramphenicol, kanamycin, nalidixic acid, erythromycin, tetracycline and metronidazoles. When caffeine was used at a concentration of 1/2MIC in combination with chloramphenicol, it was antagonistic to 9 out of 10 strains tested (two strains of *P*. *aeruginosa, Proteus vulgaris, E*. *coli, Bacillus cereus, Shigella sonnei, Plesiomonas shigellosis, K*. *pneumoniae, and Enterobacter cloacae*) and for one strain of *Enterococcus faecalis*, the interaction was indifferent. In the case of the combination of 1/2MIC caffeine with kanamycin, an antagonistic interaction was observed for one strain of *P*. *aeruginosa*, for the other strain of *P*. *aeruginosa* and *Enterobacter cloacae* an indifferent result, and for the remaining strains an additive result. Combining nalidixic acid and caffeine at a concentration of 1/2MIC, antagonism was observed in all strains except *K. pneumoniae* where the interaction was indifferent. Erythromycin, combined with the same concentration of caffeine as in the previous combinations, also produced an antagonistic interaction in most strains, where an indifferent interaction was detected for *E. coli* and one strain of *P. aeruginosa*. When combined with tetracycline, an antagonistic interaction was observed in 6 out of 10 tested strains (*Proteus vulgaris, Bacillus cereus, Shigella sonnei, Plesiomonas shigellosis, K*. *pneumoniae*, and *Enterococcus faecalis*), while indifferent interactions were detected in the remaining strains with the exception of one of the *P. aeruginosa* strains where the interaction was additive. The last antimicrobial tested in combination with caffeine at a concentration of 1/2MIC was metronidazole, and with this combination, antagonism was observed in all strains except *Bacillus cereus* and *Enterococcus faecalis*, where the interaction was indifferent. The effects of combining caffeine and antibiotics may be influenced by physicochemical interactions, complex formation, or competition for binding sites. Caffeine and aminoglycoside antibiotics, like kanamycin, target ribosomes and interfere with protein synthesis. The interaction between caffeine and kanamycin may result from their complementary activity, with caffeine increasing susceptibility to kanamycin and intercalating into DNA. However, the putative complexes formed between caffeine and erythromycin may prevent both agents from reaching their target sites of action [87].

#### 4.2.5 Other alkaloids

In a test with four antibiotics from different groups, the alkaloids tetrandrine and demethyltetrandrine isolated from *Stephania tetrandra* enhanced the inhibitory efficacy of only one of them, cefazolin [79]. Several studies suggest that sanguinarine, a benzophenanthridine alkaloid isolated from *Sanguinaria canadensis*, is antibacterial and positively interacts with other antibiotics. In a study where ampicillin, oxacillin, norfloxacin, ciprofloxacin, and vancomycin were used, sanguinarine noticeably reduced the MICs of all selected antibiotics, lowering this 32-fold in the best case. However, the best results were obtained when combining sanguinarine with one of the fluoroquinolones and showed strong synergic effects [80]. Sanguinarine also showed stronger synergistic antimicrobial activity when EDTA was added to the two-drug combination of sanguinarine and antibiotic. Two studies by Hamoud et al. [81,103] showed that a three-compound combination of sanguinarine (0.5–128 μg/mL), EDTA, and streptomycin or vancomycin had a much stronger ability to improve antimicrobial activity, probably because of the action of EDTA in disturbing the permeability of the bacterial cell wall by chelating Ca^2+^ and Mg^2+^ cations, which are essential for the wall's stability. Lysergol, a clavine alkaloid isolated from *Ipomoea muricata*, does not possess antimicrobial activity. Still, when combined with nalidixic acid, it demonstrated significant synergistic activity, at the concetration of 10 μg/mL, by inhibiting adenosine-triphosphate-dependent efflux pumps, which, as noted, are responsible for the development of MDR [82].

In research with several strains of Gram-negative bacteria, squalamine (at the concetration of MIC/10 and MIC/5) lowered the MICs of chloramphenicol, cefepime, tetracycline, and ciprofloxacin significantly against *E. coli*, *K. pneumoniae*, *P. aeruginosa*, and *Enterobacter aerogenes* strains by increasing bacterial susceptibility and drug penetration [83]. Tomatidine (4–8 mg/L) potentiates the bactericidal activity of aminoglycoside antibiotics such as gentamicin or tobramycin against *S. aureus*, and that effect was reported for several strains, including multiresistant ones. This action of tomatidine probably involves increasing the uptake of aminoglycoside antibiotics by improving the permeability of *S. aureus* [84].

### 4.3 Flavonoids

Flavonoids are a group of secondary metabolites with variable phenolic structures, and they can be found in fruits, vegetables, grains, bark, roots, stems, flowers, tea, and wine. They have shown anti-oxidative, anti-inflammatory, anti-mutagenic, and anti-carcinogenic properties [104]. In addition to these effects, studies have also shown that some flavonoids, including but not limited to diosmin, baicalein, and silibinin, are promising sources of future adjuvants in antimicrobial therapy [105]. Collected examples of tested combinations of these compounds with drugs currently used in antibacterial therapy are presented in Table 3 [[106], [107], [108], [109], [110], [111], [112], [113], [114], [115], [116], [117], [118], [119], [120], [121], [122]].

**Table 3:** Combinations of flavonoids with antimicrobial agents.

Combinations of flavonoids with antimicrobial agents	Compounds naturally occurring in plants	Antimicrobial	Bacteria species	Refs.
Combinations of flavonoids with antimicrobial agents resulting in minimum inhibitory concentration (MIC) reduction	Diosmin	Erythromycin	*Staphylococcus aureus*	[106]
		Fusidic acid	*Staphylococcus aureus*	[106]
		Gentamicin	*Staphylococcus aureus*	[106]
		Amoxicillin/clavulanic acid	*Mycobacterium marinum*	[107]
	Silibinin	Ampicillin	*Staphylococcus aureus*	[108]
			Oral bacteria	[109]
		Oxacillin	*Staphylococcus aureus*	[108]
		Gentamicin	Oral bacteria	[109]
	Baicalein	Oxacillin	*Staphylococcus aureus*	[110,111]
		Ciprofloxacin	*Staphylococcus aureus*	[110]
		Erythromycin	*Staphylococcus aureus*	[110]
		Gentamicin	*Staphylococcus aureus*	[110]
		Kanamycin	*Staphylococcus aureus*	[110]
		Fusidic acid	*Staphylococcus aureus*	[110]
		Tetracycline	*Staphylococcus aureus*	[111]
			*Escherichia coli*	[111]
		Cefmetazole	*Staphylococcus aureus*	[111]
		Ampicillin	*Staphylococcus aureus*	[111]
		Linezolid	*Staphylococcus aureus*	[112]
		Cefotaxime	*Klebsiella pneumoniae*	[113]
	Baicalin	Azithromycin	*Staphylococcus aureus*	[114]
	Licoricidin	Oxacillin	*Staphylococcus aureus*	[115]
	Quercetin	Amoxicillin	*Staphylococcus aureus*	[116]
		Ampicillin	*Staphylococcus aureus*	[116]
		Ceftriaxone	*Staphylococcus aureus*	[116]
		Cefradine	*Staphylococcus aureus*	[116]
		Methicillin	*Staphylococcus aureus*	[116]
		Cefixime	*Staphylococcus aureus*	[116]
		Imipenem	*Staphylococcus aureus*	[116]
		Sulfamethoxazole/trimethoprim	*Staphylococcus aureus*	[116]
	Morin and rutin	Amoxicillin	*Staphylococcus aureus*	[116]
		Ampicillin	*Staphylococcus aureus*	[116]
		Ceftriaxone	*Staphylococcus aureus*	[116]
		Cefradine	*Staphylococcus aureus*	[116]
		Methicillin	*Staphylococcus aureus*	[116]
		Cefixime	*Staphylococcus aureus*	[116]
		Imipenem	*Staphylococcus aureus*	[116]
		Sulfamethoxazole/trimetoprim	*Staphylococcus aureus*	[116]
	Luteolin	Ampicillin	*Staphylococcus aureus*	[117]
		Cefradine	*Staphylococcus aureus*	[117]
		Ceftriaxone	*Staphylococcus aureus*	[117]
		Imipenem	*Staphylococcus aureus*	[117]
		Methicillin	*Staphylococcus aureus*	[117]
		Erythromycin	*Listeria monocytogenes*	[118]
	Kumatakenin	Ampicillin	*Listeria monocytogenes*	[118]
			*Staphylococcus aureus*	[118]
			*Escherichia coli*	[118]
			*Pseudomonas aeruginosa*	[118]
		Erythromycin	*Listeria monocytogenes*	[118]
			*Staphylococcus aureus*	[118]
			*Escherichia coli*	[118]
		Gentamicin	*Listeria monocytogenes*	[118]
			*Pseudomonas aeruginosa*	[118]
	Apigenin	Ampicillin	*Staphylococcus aureus*	[118]
		Erythromycin	*Listeria monocytogenes*	[118]
			*Staphylococcus aureus*	[118]
			*Escherichia coli*	[118]
			*Pseudomonas aeruginosa*	[118]
		Gentamicin	*Escherichia coli*	[118]
	Quercetin	Ampicillin	*Staphylococcus aureus*	[118]
		Erythromycin	*Listeria monocytogenes*	[118]
			*Staphylococcus aureus*	[118]
			*Escherichia coli*	[118]
		Gentamicin	*Listeria monocytogenes*	[118]
			*Staphylococcus aureus*	[118]
			*Escherichia coli*	[118]
			*Pseudomonas aeruginosa*	[118]
	Chrysin	Ampicillin	*Staphylococcus aureus*	[118]
		Erythromycin	*Listeria monocytogenes*	[118]
			*Escherichia coli*	[118]
		Gentamicin	*Staphylococcus aureus*	[118]
			*Pseudomonas aeruginosa*	[118]
	Extract from plant *Adenocalymma marginatum*	Ampicillin	*Staphylococcus aureus*	[119]
			*Enterococcus faecalis*	[119]
			*Klebsiella pneumoniae*	[119]
			*Morganella morganii*	[119]
		Gentamicin	*Staphylococcus aureus*	[119]
			*Enterococcus faecalis*	[119]
			*Escherichia coli*	[119]
			*Pseudomonas aeruginosa*	[119]
			*Morganella morganii*	[119]
		Oxacillin	*Staphylococcus aureus*	[119]
			*Enterococcus faecalis*	[119]
	Extract from plant *Amphilophium vauthieri*	Ampicillin	*Staphylococcus aureus*	[119]
			*Enterococcus faecalis*	[119]
			*Enterobacter cloacae*	[119]
			*Klebsiella pneumoniae*	[119]
			*Morganella morganii*	[119]
			*Proteus mirabilis*	[119]
		Gentamicin	*Staphylococcus aureus*	[119]
			*Enterococcus faecalis*	[119]
			*Escherichia coli*	[119]
			*Klebsiella pneumoniae*	[119]
		Oxacillin	*Staphylococcus aureus*	[119]
			*Escherichia coli*	[119]
	Extract from plant *Cuspidaria convoluta*	Ampicillin	*Staphylococcus aureus*	[119]
			*Enterococcus faecalis*	[119]
			*Escherichia coli*	[119]
			*Pseudomonas aeruginosa*	[119]
			*Enterobacter cloacae*	[119]
			*Klebsiella pneumoniae*	[119]
			*Morganella morganii*	[119]
			*Proteus mirabilis*	[119]
		Gentamicin	*Staphylococcus aureus*	[119]
			*Enterococcus faecalis*	[119]
			*Escherichia coli*	[119]
			*Enterobacter cloacae*	[119]
			*Klebsiella pneumoniae*	[119]
			*Morganella morganii*	[119]
		Oxacillin	*Staphylococcus aureus*	[119]
			*Enterococcus faecalis*	[119]
	Extract from plant *Fridericia caudigera*	Ampicillin	*Staphylococcus aureus*	[119]
			*Enterococcus faecalis*	[119]
			*Escherichia coli*	[119]
			*Pseudomonas aeruginosa*	[119]
			*Enterobacter cloacae*	[119]
			*Klebsiella pneumoniae*	[119]
			*Morganella morganii*	[119]
		Gentamicin	*Staphylococcus aureus*	[119]
			*Enterococcus faecalis*	[119]
			*Escherichia coli*	[119]
			*Enterobacter cloacae*	[119]
			*Klebsiella pneumoniae*	[119]
			*Morganella morganii*	[119]
			*Proteus mirabilis*	[119]
		Oxacillin	*Staphylococcus aureus*	[119]
			*Enterococcus faecalis*	[119]
Combinations of flavonoids with antimicrobial agents resulting in MIC increase	Silibinin	Gentamicin	*Enterobacter cloacae*	[120]
	Hesperetin and naringenin	Methicillin	*Staphylococcus aureus*	[121]
		Penicillin G	*Staphylococcus aureus*	[121]
		Oxacillin	*Staphylococcus aureus*	[121]
	Quercetin	Ciprofloxacin	*Staphylococcus aureus*	[116]
		Levofloxacin	*Staphylococcus aureus*	[116]
	Morin and rutin	Ciprofloxacin	*Staphylococcus aureus*	[116]
		Levofloxacin	*Staphylococcus aureus*	[116]
	Luteolin	Ciprofloxacin	*Staphylococcus aureus*	[117]
		Levofloxacin	*Staphylococcus aureus*	[117]
	Isoquercetin	Kanamycin	*Escherichia coli*	[122]
		Amikacin	*Escherichia coli*	[122]
		Neomycin	*Escherichia coli*	[122]
		Gentamicin	*Escherichia coli*	[122]
	Apigenin	Gentamicin	*Listeria monocytogenes*	[118]

#### 4.3.1 Diosmin

Diosmin, isolated from citrus fruits and plants from the family Rutaceae and its aglycone diosmetin, has been the subject of much research on their antimicrobial activity. Diosmin proved to have strong inhibitory and bactericidal activity against the Gram-positive bacteria but not against the Gram-negative bacteria that were used in a test [106]. Diosmetin (8–128 μg/mL), in combination with erythromycin, was such a potent synergistically acting combination that it inhibited macrolide-resistant *S. aureus* growth more strongly than the positive control, verapamil. This flavonoid also improved the growth inhibitory effects of fusidic acid, gentamicin, and ciprofloxacin, but not as strongly as with erythromycin, and no interaction was observed when it was tested with kanamycin [106]. In a model in which *Drosophila melanogaster* flies were infected with *Mycobacterium marinum*, a combination of amoxicillin-clavulanic acid and diosmin (which is rapidly converted into the aglycone form diosmetin after oral administration) significantly improved the survivability of the flies. The best results were obtained from the combination of 1 mg/mL amoxicillin-clavulanic acid and 2 mg/mL diosmin, the survival outcome of which was 66.7%. The synergistic effect of the combination was especially noticeable and seen against the lack of marked improvement in the flies’ survival when treated with different concentrations of either amoxicillin-clavulanic acid or diosmin individually. The same effect was observed in an in vitro test with a multi-drug-resistant clinical isolate of *Mycobacterium tuberculosis* [107].

#### 4.3.2 Silibinin

Another flavonoid, silibinin, is a mixture of two diastereomers, silybin A and silybin B, in an approximately equimolar ratio, and it can be extracted from the seeds of the fruit of the milk thistle (*Silybum marianum*) [123]. Silibinin proved to help treat infection with both MSSA and MRSA strains and even against clinical isolates of MRSA strains. This flavonoid in concentration of 1/2MIC, in combination with ampicillin or oxacillin, gave a synergistic effect which lowered the MIC for most tested strains and the rest showed additive interaction [108]. In combination with silibinin for each strain, the MIC for ampicillin was reduced in all tested bacteria (*Streptococcus mutans*, *Streptococcus sanguinis* (*S. sanguinis*), *Streptococcus sobrinus*, *Streptococcus ratti* (*S. ratti*), *Streptococcus criceti*, *Streptococcus anginosus*, *Streptococcus gordonii*, *Aggregatibacter actinomycetemcomitans*, *Fusobacterium nucleatum*, *Prevotella intermedia* (*P. intermedia*), and *Leek phyromonas gingivalis*), producing a synergistic effect as defined by FICI ≤ 0.5. The MBC for ampicillin has shown synergistic effects in all tested bacteria expect *S. sanguinis*, *S. ratti*, and *P. intermedia*. In combination with silibinin, the MIC/MBC for gentamicin was reduced in all tested bacteria expect *S. sanguinis* and *S. ratti* [109]. In a study using another Gram-negative bacterium, *Enterobacter cloacae*, which is considered to be resistant to gentamicin, this antibiotic was effective only at concentrations well above the acceptable limit for clinical use. After combining silibinin with gentamicin, not only did the combination not show any synergistic effect, as could be expected from other studies using this flavonoid, but also, in some ratios, antagonism was observed. The extent of antagonism was higher when they were combined at a higher ratio of gentamicin,with the highest FIC value when ratio gentamicin:silibinin was 7:3. The author suggested that silibinin may antagonize gentamicin through different pathways including inhibition of its permeation into the intracellular compartment, triggering efflux of gentamicin outside the cell, enhancement of gentamicin modification, or induction of gentamicin binding to its target site of the ribosome via competitive or non-competitive binding [120].

#### 4.3.3 Baicalein

Baicalein is a flavone, originally isolated from the roots of *Scutellaria baicalensis* and *Scutellaria* *lateriflora*, it can be found in other plants like *Oroxylum indicum* (the Indian trumpet flower) and thyme. In studies, baicalein showed promising results as an additive in antibiotic therapy for various MRSA strains. The best result was obtained when baicalein (16–32 μg/mL) was combined with oxacillin against MRSA *S*. *aureus* when the FICI was 0.28, which indicates a synergistic effect. Synergy in inhibiting the growth of the bacteria was also observed with baicalein (16–32 μg/mL) and ciprofloxacin for a NorA efflux pump-overexpressing strain when the FICI outcome was 0.38. Synergistic interaction was manifested against an MRSA efflux pump-overexpressing strain in 3 out of 5 tests when baicalein (16–32 μg/mL) was combined with erythromycin. Other notable interactions were the additive effects of combining baicalein (16–32 μg/mL) with gentamicin, kanamycin, and fusidic acid against aminoglycoside-resistant strains [110]. In a study by Fujita et al. [111], an effective compound from the thyme extract was isolated and identified as baicalein. It markedly reduced the MIC of tetracycline with MRSA and *E. coli* strains, but the effect disappeared after 21 h, which probably means that this flavonoid is gradually degraded or bound to the cells of at least some strains after 21 h. Baicalein restored the effectiveness of tetracycline against MRSA and *E. coli* resistant to tetracycline strains by inhibiting the TetK tetracycline efflux pump. Besides interaction with tetracycline, baicalein noticeably reduced the MICs of β-lactams, oxacillin, cefmetazole, and ampicillin against MRSA. Surprisingly, in contrast to the finding of a study mentioned before, no interaction was observed with erythromycin or kanamycin. Moreover, baicalein (25 μg/mL) did not affect the susceptibility of MRSA to chloramphenicol or ofloxacin at all [111]. Another antibiotic useful in treating MRSA infections is linezolid, but high doses of this antibiotic may cause side effects such as dose-dependent thrombocytopenia. Baicalein, in concentration of 100 mg/kg body weight, enhanced the inhibitory effect of linezolid against biofilm-associated MRSA strains, which could reduce the dose needed to treat infections caused by these strains [112]. Baicalein (1–128 μg/mL) can suppress the messenger RNA (mRNA) expression of *CTX-M-1*, the most dominant gene for β-lactam antibiotic resistance in ESBL-positive *K*. *pneumoniae* strains. This action of baicalein is synergistic when combined with cefotaxime against some strains of this type [113]. Baicalin (baicalein 7-*O*-glucuronide) is the glucuronide of baicalein. In an in-vitro test, adding baicalin to the medium lowered the MIC of azithromycin for azithromycin-resistant *Staphylococcus saprophyticus* because the flavonoid competitively bound to the drug-binding site and inhibited ATP hydrolysis, inhibiting drug efflux from intracellular to extracellular spaces. Baicalin showed different levels of synergy in different concentrations, but the best result with a FICI value of 0.375 was obtained with baicalin at 0.25 × MIC concentration [114]. Strains of Gram-negative bacteria, the β-lactamases with broader activity and some resulting resistance to antibiotics from the β-lactam group, are becoming a growing threat. Currently, a commonly used inhibitor of β-lactamases is clavulanic acid; however, there are reports of strains also resistant to this antimicrobial [124].

#### 4.3.4 Licoricidin

Licoricidin is a member of the class of hydroisoflavanes, isolated from *Glycyrrhiza uralensis*, also known as Chinese liquorice. In combination with this isoflavane (4–8 μg/mL), the MIC of oxacillin against MRSA strains ranged between lower than 1/128 and lower than 1/1000 of the MIC in the absence of this compound. It was suspected that the mechanism responsible for this effect was the ability of licoridicin to influence protein-binding protein 2' (PBP2') formation, but studies have shown that this compound does not affect PBP2′ formation. Therefore, the mechanism by which licoricidin reduces the MIC of oxacillin still being unknown, and it is possible that this isoflavane may affect the enzymatic function of PBP2' [115].

#### 4.3.5 Quercetin

Quercetin is a pentahydroxyflavone contained in many plants, including medical, like *Ginkgo biloba*, *Hypericum perforatum*, and elderberry, but it is mainly derived from onions, apples, and tea [125].

This flavonoid was the subject of another study using more than one flavonoid in combination with an antibiotic. Antibiotics such as amoxicillin, ampicillin, ceftriaxone, cefradine, methicillin, cefixime, imipenem, and sulfamethoxazole-trimethoprim increased their activity when they were combined with quercetin or the rutin and morin pair, and the flavonoids had an additive effect. However, combining the flavonoids quercetin and the morin and rutin pair and the antibiotics cefradine, imipenem, ceftriaxone, and methicillin gave even stronger results, and the interactions manifested synergistic effects. There have been reports that phenolic compounds can diffuse through the cytoplasmic membrane of bacteria, increasing its permeability. This leads to the leakage of bacterial cell components, including potassium. A potassium leakage measurement can be used to measure cell membrane damage [126]. All tested antibiotics and flavonoids induced releases of K^+^, but a flavonoid-antibiotic combination caused more leakage than these compounds alone. These results suggested that cytoplasmic membrane damage in conjunction with cell wall damage, is the mechanism of action of these combinations of flavonoid-antibiotics. This effect was even stronger when quercetin, morin, and rutin (in concentrations: 75–600, 100–800, and 100–800 μg/mL, respectively) were combined with ampicillin, amoxicillin, ceftriaxone, cefradine, imipenem, and methicillin. Many flavonoids show synergistic effects with many different antibiotics, but unfortunately, this is not a universal rule for the flavonoid group. Antagonistic combinations include the combination of morin and rutin with ciprofloxacin or levofloxacin and the combination of quercetin with ciprofloxacin or levofloxacin. These combinations inhibited the growth of MRSA strains in smaller average zones. The antagonistic effect was even greater when a mixture of quercetin, morin, and rutin was used instead of one flavonoid [116]. Isoquercetin, a glycosylated flavonoid derived from quercetin, exhibits in subinhibitory concentration (MIC/8) an antagonistic effect in combination with the aminoglycosides kanamycin, amikacin, neomycin, and gentamicin, probably deriving from mutual chelation. Since quercetin showed no such interaction, the antagonism between this flavonoid and the antibiotics and higher MIC, when applied against the multiresistant strain of *E. coli*, may be due to glucose linked to the structure [122].

#### 4.3.6 Luteolin

Luteolin (500 μg/mL), a flavonoid obtained from the plant *Reseda luteola*, exerted an additive effect on ampicillin, cefradine, ceftriaxone, imipenem, and methicillin, with the greatest decrease in MIC observed in combination with imipenem. Adding quercetin enhanced the positive effect of luteolin on antibiotics, resulting in improved and synergistic interactions with ceftriaxone and imipenem. These flavonoids can also deplete intracellular K^+^, compromising bacteria cells' integrity and viability by inducing cytoplasmic membrane damage. Again, a combination of more than one flavonoid, in this case, luteolin and quercetin, resulted in a greater synergistic interaction than using just one of these flavonoids [117].

#### 4.3.7 Hesperetin and naringenin

As may be seen, many flavonoids show synergistic effects with many different antibiotics, but beneficial adjuvanticity is not a given for all of them. Hesperetin and naringenin are flavonoids in citrus fruits such as grapefruits and lemons. Previous studies have shown that flavonoids usually have synergistic or no interaction with β-lactams, hesperetin, and naringenin have demonstrated antagonism in interactions with this type of antibiotics. Combining these two flavonoids, at their inhibitory concentration (for hesperetin 250 and 500 mg/mL and for naringenin 125 and 250 mg/mL for MSSA and MRSA strains, respectively), and one of the tested β-lactams (methicillin, penicillin, or oxacillin) against MRSA and MSSA strains gave the same result: the complete cancellation of any effect of either group of compounds in the bacterial growth environment. The exception, however, was cefoxitin, which did not block all effects of flavonoids in this environment. These flavonoids also showed no effect on β-lactamase enzymes or PBP2’ levels compared to the control [121].

#### 4.3.8 Plant extracts containing flavonoids

An extract of *Buddleja albiflora*, a plant native to the mountains of central China, contained four flavonoids that exhibited antimicrobial activity: apigenin chrysin and kumatakenin. Even though all three compounds showed activity against the tested strains of *L. monocytogenes*, *S. aureus*, *E. coli*, and *P. aeruginosa*, only kumatakenin showed synergistic interaction with all three tested antibiotics, erythromycin (kumatakenin concertation of 64 μg/mL), gentamicin (kumatakenin concertation of 64 μg/mL), and ampicillin (kumatakenin concertation of 32 μg/mL), against *L. monocytogenes*. Apigenin (32 μg/mL) and chrysin (256 μg/mL) were also able to demonstrate synergistic interactions, but only with erythromycin against *P. aeruginosa* and *L. monocytogenes,* respectively. In other combinations, the interaction was only additive, none occurred, or it was antagonistic, in the last case, only in the combination of apigenin and gentamicin. In this study, not only the antimicrobial activity but also the antibiofilm abilities of synergistic compounds were tested. Again, kumatakenin yielded the best results and was able to enhance the antibiofilm potential of all antibiotics and make *L. monocytogenes* biofilm more sensitive to erythromycin, gentamicin, and ampicillin [118]. Other plants that transpired to contain antibacterial flavonoids were from the family *Bignoniaceae*, and more specifically, the *Adenocalymma marginatum*, *Amphilophium vauthieri*, *Cuspidaria convoluta*, and *Fridericia caudigera*. Four extracts were isolated from them, in which active compounds were identified. Their influence on ampicillin, gentamicin and oxacillin activity against *Staphylococcus* strains and Gram-negative bacteria was examined. The compounds of interest were apigenin, chrysin, and luteolin, and they were identified by comparing retention times and the ultraviolet-visible (UV-vis) spectrum and then confirmed by high-performance liquid chromatography-tandem mass spectrometry (HPLC-MS/MS). Overall, the combinations of all tested antibiotics and all tested extracts showed enhanced antimicrobial effects against almost all Gram-positive strains, and the same results were observed with the MICs of ampicillin and gentamicin against some Gram-negative strains. However, the best results were obtained when combined with ampicillin and *Cladonia* *convoluta* extract. The *Fridericia* *caudigera* extract also showed good results, revealing the best activity in combination with antibiotics against *Staphylococcus* strains and the best enhancement of the action of oxacillin [119].

### 4.4 Tannins

Tannins are a group of bitter and astringent substances derived from phenolic acids and classified as phenolic compounds. They were originally used in the leather production industry in the tanning of animal hides, but after much research, tannins are now known to be potent antioxidants and be cardioprotective, anti-inflammatory, anticarcinogenic, antimutagenic, and antimicrobial [127,128]. The interactions between tannins and antibacterial drugs are summarized in Table 4 [[129], [130], [131], [132], [133], [134], [135], [136]].

**Table 4:** Combinations of tannins with antimicrobial agents.

Combinations of tannins with antimicrobial agents	Compounds naturally occurring in plants	Antimicrobial	Bacteria species	Refs.
Combinations of tannins with antimicrobial agents resulting in minimum inhibitory concentration (MIC) reduction	Corilagin	Oxacillin	*Staphylococcus aureus*	[129,130]
		Cefmetazole	*Staphylococcus aureus*	[129]
		Imipenem	*Staphylococcus aureus*	[129]
		Benzylpenicillin	*Staphylococcus aureus*	[129]
	Tellimagrandin I	Oxacillin	*Staphylococcus aureus*	[130]
	Tellimagrandin II	Oxacillin	*Staphylococcus aureus*	[131]
		Doxycycline	*Staphylococcus aureus*	[131]
	Punicalagin	Oxacillin	*Staphylococcus aureus*	[132]
	Tannic acid	Oxacillin	*Staphylococcus aureus*	[133]
		Fusidic acid	*Staphylococcus aureus*	[134]
		Cefotaxime	*Staphylococcus aureus*	[134]
		Minocycline	*Staphylococcus aureus*	[134]
		Rifampicin	*Staphylococcus aureus*	[134]
		Vancomycin	*Staphylococcus aureus*	[134]
		Ofloxacin	*Staphylococcus aureus*	[134]
	Gallic acid	Fusidic acid	*Staphylococcus aureus*	[134]
		Minocycline	*Staphylococcus aureus*	[134]
		Cefotaxime	*Staphylococcus aureus*	[134]
		Rifampicin	*Staphylococcus aureus*	[134]
		Gentamicin	*Escherichia coli*	[135]
		Sulfamethoxazole	*Escherichia coli*	[135]
		Amikacin	*Escherichia coli*	[135]
		Norfloxacin	*Escherichia coli*	[135]
Combinations of tannins with antimicrobial agents resulting in MIC increase	Gallic acid	Carbenicillin	*Chromobacterium violaceum*	[136]
		Tetracycline	*Chromobacterium violaceum*	[136]
	Tannic acid	Carbenicillin	*Chromobacterium violaceum*	[136]
		Tetracycline	*Chromobacterium violaceum*	[136]

#### 4.4.1 Corilagin

Corilagin is an ellagitannin and a major active component of many plants such as *Phyllanthus niruri* L., *Phyllanthus emblica* L., and *Phyllanthus urinaria* L.. [137].

Corilagin alone showed weak activity against MRSA strains, but at a much weaker concentration than the MIC (16 μg/mL), it markedly decreased the MIC of oxacillin and other tested β-lactams (cefmetazole, imipenem, and benzylpenicillin). The potency of corilagin and oxacillin against MRSA was proved by the FIC of 0.13, which means a synergistic effect. Corilagin did not show such remarkable effects against MSSA strains when the FIC was 0.75, which means an additive effect. This difference may indicate that corilagin inhibits PBP2' in MRSA cells, known as a methicillin-resistance determinant, but not other PBPs in MSSA strains [129]. PBPs can transfer resistance to β-lactam antibiotics in bacterial cells. In another study, where not only corilagin was tested but also another tannin, tellimagrandin I, the mechanism of inactivation of PBPs (especially PBP2a) by these tannins was demonstrated in an in-vitro test, in which MRSA strains were grown in the presence of corilagin and tellimagrandin I completely lost the ability to bind to fluorescent-labelled benzylpenicillin. This inhibition of PBP activity would restore β-lactams’ antibacterial effect on MRSA, and that was proved in a significant reduction in the MICs of oxacillin when it was in interaction with corilagin (16 μg/mL) and tellimagrandin I (50 μg/mL). Interestingly, their hydrolyzed products, ellagic acid and gallic acid, did not affect the MIC of this β-lactam, which is important because corilagin and tellimagrandin I are hydrolyzable tannins which tend to be hydrolyzed by acid or enzymes to glucose, ellagic acid, and gallic acid [130].

#### 4.4.2 Tellimagrandin I

Tellimagrandin I, an ellagitannin found in plants such as *Cornus canadensis*, *Eucalyptus globulus*, or *Rosa rugosa*, did not show a significant effect against MRSA strains when it was used alone. However, adding this tannin, in concentration 50 μg/mL, to the medium significantly reduced the MIC of oxacillin. This combination showed a synergistic effect (FIC = 0.39) but was not as strong as the effect of a combination of corilagin with the antibiotic when the FIC was 0.13. Tellimagrandin I also greatly reduced the MICs of other β-lactams, such as benzylpenicillin and ampicillin, against MRSA strains [130].

#### 4.4.3 Tellimagrandin II

Another ellagitannin is tellimagrandin II, which can be extracted from the shells of *Trapa bispinosa*, also known as the water chestnut. This tannin in concentration of 40 μg/mL significantly reduced the MIC of oxacillin and doxycycline against MRSA strains [131]. The values of FIC indicated synergistic effects with both doxycycline and oxacillin against almost all tested strains. The mechanisms of tellimagrandin II possibly responsible for the appreciable decrease in the MIC are the inhibition of mecA transcription and reduction of the penicillin-binding of PBP2a, which affect the synthesis and function of resistance-deprived proteins in MRSA. Tellimagrandin II could disrupt bacterial morphology and cause cell plasmolysis [131].

#### 4.4.4 Punicalagin

Punicalagin is one of the main tannins isolated from *Punica granatum*. The presence of this compound in concentration 1/2MIC (31.25 μg/mL) was able to reduce the MIC of oxacillin 4- to 64-fold against MRSA. The increase in the susceptibility of MRSA to oxacillin mediated by punicalagin may be due to the reduced transcription of mecA, which resulted in reduced PBP2a levels; as mentioned before, PBP2a is a methicillin resistance determinant [132].

#### 4.4.5 Tannic acid

Tannic acid is a tannin naturally occurring in practically all aerial plant tissues. This compound was historically used for treating diarrhoea, topically dressing burns, and rectally treating unspecified disorders [138]. In research, this tannin showed antibacterial action because using tannic acid (100 mg/L) in Mueller-Hinton agar and Mueller-Hinton agar with 10% rabbit plasma decreased the MIC of oxacillin to ≤0.06 mg/L. Tannic acid was also able to inhibit plasma coagulation by *S. aureus* at a concentration that was below the MIC. This inhibition was probably caused by a weakening of ionic calcium concentration due to the chelation ability of tannic acid, suppressing enzyme production and enzyme reaction [133]. In another study, tannic acid was also active against MRSA with other antibiotics. This tannin in concentration of 1/4MIC, reduced the MIC of fusidic acid, cefotaxime, minocycline, rifampicin, vancomycin, and ofloxacin when it showed synergism with the first four with FICI < 0.5, and an additive effect with the other two with FICI < 1 against some strains [134].

#### 4.4.6 Gallic acid

Gallic acid is a phenolic compound, which can be found in its free state, but also as a constituent of certain tannins, namely gallotannins [139]. Gallic acid has been reported in several plants, including *Allanblackia floribunda*, *Garcinia densivenia*, *Bridelia micrantha*, *Caesalpinia sappan*, *Dillenia indica*, *Diospyros cinnabarina*, *Paratecoma peroba*, and *Terminalia bellirica*. Gallic acid has also been found to be an element of some beverages, e.g., red and white wine [140,141].

The ethyl ester of gallic acid (concentration of 1/4MIC) imparted additive effects against MRSA strains with all but one of the antibiotics tested (fusidic acid, minocycline, cefotaxime, and rifampicin), the exception being vancomycin with which it showed no effect [134]. Besides caffeine, gallic acid is a compound in high concentrations (3.5 mg/dL) in black tea. Adding 1.25 mg of the extract from black tea to antibiotic discs of gentamicin, sulfamethoxazole, amikacin, and norfloxacin suppressed the antibacterial effects of all four against *E. coli*.. When 2.5 mg of extract was used, this inhibitory effect was reduced somewhat in combination with norfloxacin and sulfamethoxazole, but still, the diameters of the bacterial growth inhibition zones were smaller than the zone diameters observed with antibiotics alone. With gentamicin, the diameter was almost equal to the diameter of the standard disc, while for amikacin, the diameter was larger than that of the standard disc. It is worth mentioning, however, that tea also contains other compounds such as caffeine, epigallocatechin, and epigallocatechin gallate (in concentrations 30.9, 0.43, and 0.25 mg/dL, respectively), which may also affect how antibiotics work. Moreover, more promising results were obtained using gallic acid not extracted from black tea. Gallic acid (5 μg/mL) alone was synergistic with all the antibiotics, and it was even more effective in higher doses (10 and 25 μg/mL), and this effect was more prominent with amikacin and sulfamethoxazole [135].

Tannic acid and gallic acid alone were also tested against *Chromobacterium* *violaceum* (*C. violaceum*), and gallic acid proved to have a weaker effect on the inhibition of the growth of this bacterium than tannic acid because only the maximum tested concentration of 2000 mg/mL of gallic acid exhibited an inhibitory effect. In comparison, tannic acid was effective at a MIC of 250 μg/mL. Both of these compounds exerted a synergistic or additive effect on some of the antibiotics in the studies cited, but when their effects were tested in combination with carbenicillin or tetracycline, the results differed from those achieved with the other drugs. The MICs of both antibiotics were increased in combination with both tannic acid and gallic acid. The MIC of carbenicillin was increased from 12 to 50 mg/mL in the presence of either of the acids, and the MIC of tetracycline rose from 15 to 30 μg/mL in combination with tannic acid and from 15 to 125 μg/mL used with gallic acid. Authors hypothesize that the reduction in antibacterial activity could be due to 1) binding of the antibiotic to tannins, 2) competitive binding of the tannins to -lactamase (in case of CAR) or similar enzymes responsible for antibiotic hydrolysis, or 3) tannins causing a modification of the bacterial properties such as biofilm formation and/or secondary metabolite synthesis, rendering them more protected to the effects of antibiotics. An important aspect of the pathogenicity of *C. violaceum* is its ability to form a biofilm. Antibiotic carbenicillin alone effectively inhibited the biofilm of *C. violaceum* at concentrations that did not prevent bacterial growth, but in the presence of a sub-MIC concentration of tannic acid or gallic acid, an enhancement of biofilm formation was observed. An interesting observation is that when tannic acid was used in a concentration below the MIC in combination with tetracycline in a sub-MIC concentration, it effectively prevented biofilm formation, even though this combination required a higher MIC of the antibiotic for a bactericidal effect. The same effect was observed in a mixture of gallic acid and carbenicillin, but only at certain higher concentrations of gallic acid. These results indicate that some of the combinations of these compounds can be useful in inhibiting biofilm formation; however, they need to be used with caution to prevent an effect opposite to desired [136].

### 4.5 Sulfoxides (allicin)

Sulfoxides are oxidized derivatives of sulfides, and they are organosulfur compounds containing a sulfinyl (>SO) functional group attached to two carbon atoms [142]. An example of an important sulfoxide is dimethyl sulfoxide (DMSO), a common solvent; however, allicin is the most significant compound in the context of antibacterial activity.

Allicin is a sulfoxide produced in garlic from alliin by a reaction using the enzyme alliinase triggered by cell damage in the clove. Allicin is also one of the main active compounds of garlic and potentially has some features beneficial to health, such as raising the immune system's efficiency [143]. Garlic has been used in treating various ailments for centuries, and the antibacterial properties of allicin have been repeatedly confirmed, but high MIC values exclude it from use alone in treating bacterial infections. However, the possibility of using this compound in combination with those preparations already in use has not been overlooked. The effect of allicin has been investigated in combination with Gram-positive and Gram-negative bacteria, and promising results were obtained for both groups, which are presented in Table 5 [[144], [145], [146], [147], [148]]. However, no information was found on the potential cases of increased MIC in combination of allicin with antibiotics.

**Table 5:** Combinations of allicin with antimicrobial agents resulting in the minimum inhibitory concentration (MIC) reduction.

Antimicrobial	Bacteria species	Refs.
Cefoperazone	*Pseudomonas aeruginosa*	[144]
Oxacillin	*Staphylococcus aureus*	[144]
	*Staphylococcus epidermidis*	[144]
Cefazolin	*Staphylococcus aureus*	[144]
	*Staphylococcus epidermidis*	[144]
Azithromycin	*Bacillus subtilis*	[145]
	*Escherichia coli*	[145]
Levofloxacin	*Bacillus subtilis*	[145]
	*Escherichia coli*	[145]
Rifampicin	*Bacillus subtilis*	[145]
	*Escherichia coli*	[145]
Ciprofloxacin	*Bacillus subtilis*	[145]
	*Escherichia coli*	[145]
	*Staphylococcus aureus*	[146]
Oxytetracycline	*Bacillus subtilis*	[145]
	*Escherichia coli*	[145]
Ornidazole	*Bacillus subtilis*	[145]
	*Escherichia coli*	[145]
Vancomycin	*Enterococci* spp.	[147]
	*Klebsiella pneumoniae*	[148]
	*Staphylococcus aureus*	[146]
Cefoxitin	*Staphylococcus aureus*	[146]
Erythromycin	*Staphylococcus aureus*	[146]
	*Staphylococcus sciuri*	[146]
Clindamycin	*Staphylococcus aureus*	[146]
Piperacillin/tazobactam	*Escherichia coli*	[146]
	*Klebsiella pneumoniae*	[146]
	*Acinetobacter baumannii*	[146]
Ceftriaxone	*Escherichia coli*	[146]
Imipenem	*Escherichia coli*	[146]
	*Klebsiella pneumoniae*	[146]
	*Pseudomonas aeruginosa*	[146]
Amoxicillin/clavulanic acid	*Escherichia coli*	[146]
	*Klebsiella pneumoniae*	[146]
	*Salmonella enteritidis*	[146]
Meropenem	*Klebsiella pneumoniae*	[146]
Gentamicin	*Staphylococcus aureus*	[146]
	*Salmonella enteritidis*	[146]
Colistin	*Pseudomonas aeruginosa*	[146]
Amikacin	*Pseudomonas aeruginosa*	[146]
Tigecycline	*Acinetobacter baumannii*	[146]

One of the species tested was *P*. *aeruginosa*, challenging which was the combination of allicin (in concentrations from 1/8MIC to 1/2MIC) with the β-lactam cefoperazone. For both susceptible and resistant strains, the FICs signified synergistic or (only in some cases) additive interactions borne out by FICs ranging between 0.28 and 0.56. When allicin was used against *Staphylococcus* sp. bacteria, it showed synergistic effects with β-lactams. For the species *S. aureus*, allicin, in combination with cefazolin, resulted in a 126-fold decrease in the MIC_90_ value, while for oxacillin, a 64-fold decrease was observed. In the case of *Staphylococcus epidermidis*, a synergistic effect was also observed for allicin combined with cefazolin and oxacillin, but a weaker effect than for *S. aureus*, as the decreases were 4- and 32-fold, respectively. For both bacterial species, all FIC values were ≤ 0.5, confirming the synergy of the observed interactions [144].

Gram-positive *Bacillus subtilis* and Gram-negative *E*. *coli* were sensitive to the action of allicin. Azithromycin, levofloxacin, rifaximin, ciprofloxacin, and oxytetracycline synergized (FIC ≤ 0.5) for both bacteria and only with ornidazole was it in an additive interaction. It is worth noting that allicin not only enhanced the antimicrobial effect of these antibiotics but also diminished their protein denaturation potential, and Allicin was thereby able to lower their dose-dependent toxicity [145].

The antibacterial effect is demonstrated not only by allicin (8–16 μg/mL) itself but also by garlic (*Allium sativum* L.) (1000–2000 μg/mL) which contains allicin in its extracts. When not only allicin but also fresh Spanish garlic (which was peeled, mashed, filtered, and freeze-dried) was mixed with vancomycin, both combinations showed synergistic effects (FIC < 0.5) against most tested vancomycin-resistant enterococci (VRE) [147]. The mechanism responsible for this synergy is the reactivity of allicin with thiol groups and its ability to form an S-allyl-mercapto adduct with them, and this leads to the oxidation and inhibition of many important thiol-containing enzymes [149]. Allicin may bind to certain enzymes in VRE on the Tn1546 transposon that codes for vancomycin resistance and, consequently, by binding with the enzymes, inhibit the transposon's activity and increase the susceptibility of *Enterococcus* sp.. This theory is confirmed by the prevention of synergistic interactions noted with cysteine, which reacts with allicin through the SH group, and with the use of β-mercaptoethanol, which breaks disulfide bonds [147]. Aqueous garlic extract (AGE) was made out of 5 g of dried garlic that was mercated with water filtered and then dried and dissolved in nutrient broth to obtain desired concentration. This extract also showed antimicrobial properties, as did garlic alone and allicin: the combination of AGE (in concentration of 62.5 μg/mL, which was lower than its MIC) and vancomycin gave a stronger antibacterial effect against *K*. *pneumonia* than when used alone. The greater effect was notable in the inhibition of bacterial growth at lower concentrations when these compounds were combined than their MICs when used separately [148].

It is important to consider how different the effects may be, which are produced by the same compound in combination with different antibiotics and tested against different species or even strains of bacteria. Ethanol garlic extract (EGE), in subinhibitory concentrations, used to challenge four Gram-positive and five Gram-negative bacteria species, gave completely different results with each species. To obtain this extract, 5 g of garlic cloves were chopped and homogenized in 10 mL of 96% ethanol, then filtered to obtain an ethanol extract with a concentration of 500 mg garlic/mL. Based on the relationships of the diameters of the growth inhibition zones among Gram-positive bacteria, the best results were shown by EGE on *S. aureus* when it interacted synergistically with cefoxitin, gentamicin, erythromycin, clindamycin, vancomycin, and ciprofloxacin and failed to interact only with penicillin. The situation was quite different in the case of *Staphylococcus* *sciuri*, where compared to *S. aureus,* against which 6 out of the 7 tested combinations showed synergism; against this bacterium, synergism occurred only in the combination of EGE and erythromycin. In the cases of Gram-negative bacteria, there were no such large differences between species. The most synergistic interactions occurred against *E. coli* and *K*. *pneumoniae*, obtained between EGE and 4 out of the 7 antibiotics tested (piperacillin/tazobactam, imipenem, meropenem, amoxicillin/clavulanic acid against *K*. *pneumoniae* and piperacillin/tazobactam, ceftriaxone, imipenem, and amoxicillin/clavulanic acid for *E*. *coli*), and the fewest against *A*. *baumannii* in 2 out of the 7 antibiotics and EGE combinations (piperacillin/tazobactam and tigecycline) [146].

### 4.6 Mycotoxins

Different fungi naturally produce mycotoxins and are typically found in foods such as cereals, dried fruits, nuts, and spices. They are a major problem in animal feed, confirmed by a study on 74,821 samples from different countries: 88% contained at least one mycotoxin [150]. Ingestion of mycotoxins can have many negative effects, mainly on the intestine or immune system [151]. These changes may affect the action of antibacterial compounds by altering their absorbability. The number of studies focusing on interactions between mycotoxins and antibacterial compounds is low. There is a grave lack of publications examining interactions from a microbiological point of view, and accordingly, there is little research focusing on potential changes in the MIC of antibacterial substances.

#### 4.6.1 T-2

T-2 is a trichothecene mycotoxin, which occurs naturally as a product of *Fusarium* spp. and which is toxic to humans and animals. Chronic exposure to this mycotoxin caused changes in the pharmacokinetics of chlortetracycline in pigs. In samples taken from pigs receiving feed contaminated with 111 μg/kg of T-2 for 21 days, a significantly higher area under the plasma concentration-time curve and maximal plasma concentration of chlortetracycline could be observed than in samples from pigs receiving uncontaminated feed [152]. In another study, where the transepithelial passage was tested, a cytotoxic concentration of 10 ng/mL of this mycotoxin increased the passage of doxycycline and paromomycin. This is probably also due to the decrease in transepithelial electrical resistance (TEER) observed in this study. The IPEC-J2 cell line obtained from jejunum epithelia from a neonatal, unsuckled piglet was used in this study [153].

#### 4.6.2 Deoxynivalenol

Deoxynivalenol (DON), also known as vomitoxin, is a trichothecene mycotoxin produced by *Fusarium* spp.. In the previously mentioned work, apart from mycotoxin T-2, the effect of deoxynivalenol was also tested, and similarly to T-2, at a cytotoxic concentration of 5 μg/mL, it also increased the passage of doxycycline and paromomycin and also decreased TEER [153]. The IPEC-J2 cell line was also used in a study of the effect of DON on fosfomycin penetration. This study showed that the presence of 1 μg/mL of DON resulted in decreased maximum serum concentration (*c*_max_) and time to peak drug concentration (*t*_max_) for fosfomycin, which means that the tested non-toxic concentration of DON interferes with the pharmacokinetics of this antibiotic after short-term exposure [154]. Valuable data on the interaction of mycotoxins with antibiotics was also obtained in a study using jejunal explants from adult pigs, which were incubated in a buffer containing 30 μg/mL of DON. As found in the previously mentioned research, a fall in TEER for the samples incubated with DON was also demonstrated in this experiment. This mycotoxin also intensified the transport of the transcellular markers Lucifer yellow and mannitol. DON also induced the antibiotic pharmacokinetic change: it accelerated the transport of doxycycline by a factor of approximately five, which was probably caused by the ability of DON to inhibit the efflux transport of doxycycline to the intestinal lumen [155].

## 5 Conclusion

Because of the growing threat of multi-drug-resistant bacteria, searching for new therapeutic regimens is necessary for human and animal health. The fact that entirely new class has not been introduced into treatment in many years, only emphasizes how difficult it is now to develop compounds capable of bypassing the defence mechanisms developed by present microorganisms. Given the ability of bacteria to adapt and develop newer and newer resistance mechanisms, new approaches are needed to design new methods of treating infections. This review shows the great potential of naturally occurring compounds to improve the antibacterial effect of drugs currently used in medicine. Many candidates are from terpenes, alkaloids, flavonoids, or tannins. It is also worth noting that better results were obtained with combinations of these compounds, which may also indicate interactions between them and antimicrobials and interactions between these natural products. It is necessary to understand the mechanisms responsible for these interactions in more detail. The most visible in the collected publications was the ability to inhibit the bacterial efflux pump, but some compounds such as capsaicin, piperine, or mycotoxins can increase the absorption of antimicrobials. On the other hand, certain combinations impair the ability of antibacterial preparations to fight infections. These interactions may cause therapy failure or require administering a higher drug dose, which is not desirable in either human or animal medicine.

Further, more detailed studies are necessary before introducing these compounds into a standard, safe therapy used in medicine. Studies using both animals and humans are needed to verify that the results of in-vitro studies correspond to the effects obtained in vivo. The toxicity of the presented combinations and their effect on the beneficial normal microbiota should also be investigated. Standardizing the methods of determining interactions is also necessary, making these results easier to interpret and compare. Overall, despite the amount of work and research still needed to introduce plant-derived compounds into antimicrobial therapy, the endeavour is worthwhile because the outcome may herald a new era in treating bacterial infections.

## CRediT author statement

**Izabela Malczak:** Conceptualization, Investigation, Writing - Original draft preparation, Reviewing and Editing, Visualization; **Anna Gajda:** Conceptualization, Writing - Reviewing and Editing, Supervision.

## Declaration of competing interest

The authors declare that there are no conflicts of interests.
